# Bromide of Potassium in Uterine Fibroids

**Published:** 1871-05

**Authors:** 


BROMIDE OF POTASSUM IN UTERINE FIBROIDS.—In the Boston Gynæcological Society a case of fibroid tumor of the uterus, which had been as large as a "small child," was reported cured by the steady employment of bromide of potassium for eighteen months. Dr. Storer had used both this salt and the chloride of ammonium—the latter in some cases with benefit, though he seemed to think that the occasional shrinkage and disappearance of such tumors, previous to the passage of the climacteric, were rather coincidences than the effects of treatment. Dr. Sullivan preferred the bromide to iodide, because it "produced a more profound effect on the nervous system, and hastened molecular metamorphosis."—*Pacific Medical Journal.*

